# The Evolution of Long-term Care Programs: Comment on "Financing Long-term Care: Lessons From Japan"

**DOI:** 10.15171/ijhpm.2019.79

**Published:** 2019-10-28

**Authors:** Pamela Nadash

**Affiliations:** Department of Gerontology, John W. McCormack Graduate School of Policy Studies, University of Massachusetts, Boston, MA, USA.

**Keywords:** Long-term Care, Social Insurance, Aging, Entitlements, Cash for Care, Caregiver Benefits

## Abstract

The need for long-term care (LTC) represents a "new social risk," one that overlaps with and complements systems of care that pre-date such programs, complicating LTC program design. This commentary expands on Ikegami’s discussion of how these structural factors must be accommodated, as well as historical and cultural factors that influence public expectations of such a program. The commentary specifically focuses on the role of cash payments, caregiver benefits, and the sometimes indistinct line between LTC and health systems. The experiences of countries operating LTC program in a wide range of contexts can illuminate common challenges, as well as some potential solutions to these vexing design and operational issues.

Long-term care (LTC) is a relatively new public and health policy concern, one that results from successes in improving population health as well as declines in fertility across industrialized nations. Because of its relative newness, government efforts to respond to the growing demand for LTC must necessarily build on existing systems, fundamentally shaping the issues that arise.

Indeed, in many cases, a government’s need to act is based on pressures that the demand for LTC care places on existing public programs: Ikegami notes how overutilization of Japan’s hospitals by older people (and the associated pressure on healthcare costs) was a key factor behind the introduction of its LTC insurance (LTCI) program.^1^ The adoption of Germany’s program was due, in part, to cost pressures on the social assistance program financed by regional government, which funds nursing home stays for the poor.^2,3^ While any public program must build on and co-exist with established systems, LTC is particularly tricky because it overlaps with so many other policy areas: most obviously, a country’s healthcare system, but also with housing, labor, retirement, and family policy, so that boundaries between programs differ from country to country. Moreover, because so many of these areas are influenced by contentious and shifting societal norms – particularly around the role of the family generally and women specifically – the design of LTC programs can be difficult to negotiate.

This unavoidable variation across countries – in how a society sets goals for its LTC program and how it delineates LTC – reinforces Ikegami’s point about the difficulty of comparing programs. For example, a key difference lies in the role of family caregivers, and the extent to which cash payments are intended to pay for their work. Ikegami includes allowances to family caregivers as a LTC expense, reasoning that they are equivalent to cash payments. While care allowances are certainly meant to compensate carers for their work, it not clear that cash payments are primarily meant to benefit family caregivers.

Indeed, the goal of cash payments differs across countries. For example, in the US’s Medicaid program (the largest payer of LTC in the country, funding LTC on a means-tested basis), most states’ cash for care programs ban payments to spouses, and often ban payments to family members living with the care recipient. Even where permitted, not all care recipients choose to use cash payments to hire relatives. In other countries as well, cash payments are used to hire independent workers. Indeed, in some cases the political rationale for cash payments is to help develop and recruit a professional workforce. In contrast, feminists in Japan campaigned against including a cash benefit in the Japanese program, on the grounds that it would consolidate the role of women in providing LTC (a concern also in South Korea and Taiwan) – clearly assuming that a cash payment would be used to compensate family caregivers. These differences reflect the varying meanings of cash payments: whether they aim to benefit care recipients, enabling them to operationalize choice in who provides care (its predominate meaning in countries that fall into the Anglo-liberal welfare state category^4^), only indirectly benefitting family caregivers, or whether they act as family income supplements, meant to compensate carers for the work they do (as it is in Germany – although Germans, too, use cash payments to hire independent workers as necessary).

However, benefits for carers fit awkwardly with many countries’ LTC programs, often constituting a separate category and funded through separate payment streams – often, because they cover situations other than eldercare. (Many such policies focus on childcare.) While benefits such as respite fall clearly into the LTC bucket, other caregiver benefits are harder to classify. For example, some US states have introduced paid family leave (which provides income to those caring for disabled or elderly persons as well as for children), typically financed out of payroll taxes. In Germany, however, the public LTCI program finances unemployment, health insurance, and LTCI contributions for carers as well as contributions to Germany’s statutory pension fund, and paid leave is taken as a personal loan. If, however, a carer leaves employment, the LTCI program’s cash benefit can become a family income supplement, underlining how a cash benefit is fungible in some countries, but not where there are strict controls over their use.^5,6^

The case of caregiver benefits highlights the difficulty of distinguishing LTC from other services and programs. Ikegami notes another example: nursing homes, an issue that arises due to the increasingly severe health status of nursing home residents. Worldwide, countries are striving to limit nursing home admission to people with more serious health conditions, and to provide most LTC in community-based settings – ideally, in people’s own homes. Rising acuity levels among nursing home residents have raised the cost of nursing homes and, in turn, created incentives to limit access. More importantly, they have blurred the distinction between the types of care delivered in nursing homes and in other settings that traditionally fall in the health sector, such as post-acute/rehabilitative and long-term chronic care. Concurrently, such shifts have created a need to find ways of serving significantly disabled individuals in community-based settings; delivering health-related services in those settings; and ensuring quality and coordination for those services. One of the Dutch program’s recent reforms was to acknowledge this distinction, formally shifting the costs of home nursing out of the LTC program and into the health sector. However, this shift, along with other changes that split responsibility for LTC, has created its own problems: namely, poor coordination across entities and the creation of incentives to cost-shift.^7^

The Dutch program illustrates some issues that can arise out of a poorly-defined program, leading to high costs: the Dutch spent roughly 3.7 % of gross domestic products on LTC in 2015, compared to the Organisation for Economic Co-operation and Development average of 1.7%.^8^ This can be attributed to their early adoption of social insurance for LTC, back in 1968,^9^ with an initial, problematic design: the program covered a broad range of risks; utilized nursing homes heavily; had porous boundaries with other programs; and had high premiums from the very start. The program’s name – the Exceptional Medical Expenses Act (AWBZ), now the Long-term care Act (Wlz) – hints at how broad its initial goals were: in addition to explicitly covering LTC, it covered rehabilitation, hospital stays of over a year, and mental health care. The program also covered people of all ages. Thus, a major cause of budget pressures resulted from demand from children with learning disabilities, autistic spectrum diagnoses, and intellectual disabilities who otherwise lacked services. To solve these problems, a 2015 reform re-allocated program responsibilities among several different programs, leading to the aforementioned concerns about increasing fragmentation and cost-shifting.

This potted history of the Dutch program aims to highlight the importance of defining the core constituency of a LTC program and the needs that a program is aiming to address – as well as the influence that other areas of health and social policy can have on LTC programs. A key cost-control component of the Japanese program is that is it limited to older people and those with specified “geriatric diseases,” an approach that South Korea has copied.^10^ The German program, in contrast, covers people of all ages, and recently revised its eligibility assessment to more explicitly cover people with dementia.^11^ Indeed, many LTC programs cover people of all ages. The downside of a unified program is that it can create a large political constituency supporting expanded benefits (leading to expanded costs); the differing political constituencies may also have significantly different goals. On the other hand, separate programs can create discontinuities and inequalities across populations. For example, Australia operates a generous LTC program for younger people with disabilities and a less generous aged care program, creating problems as people transition from one to the other. However, other political issues can arise if it is perceived that benefits received by older people are at the expense of younger or future generations, an issue that Japan has addressed by limiting the contribution base for its LTCI program to people 40 or older.

Although LTC’s emergence as a “new social risk”^12^ is complicated by its need to complement existing systems, the field has benefitted considerably from cross-national learning. Germany purposefully incorporated cost control mechanisms into its design. Japan built on the German program, while South Korea, in turn, learned from Japan’s experience; notably, it intentionally started small,^13^ as Ikegami recommends, with strict eligibility criteria and comparatively low contribution levels. Taiwan, too, has carefully studied other countries’ design in developing its yet-to-be-launched program, although, interestingly, it has seen a backlash against the social insurance model recommended by its policy experts,^14,15^ – a development that should remind the reader that local politics and history will nearly always trump careful planning. History also affects what citizens feel is owed them, which in turn influences how a program evolves. Although social insurance models are critiqued for creating a sense of entitlement among citizens, entitlement can arise from historical or social factors, as the evolution of Japan’s program demonstrates.

Ikegami concludes by advising countries to start early in developing their LTC programs, so they can start small and constrain expectations – good advice, certainly. However, things rarely goes as planned: gestation periods for LTC programs can be extremely long. While researchers and policy experts—not to mention ordinary people—understand the critical and pressing need for LTC programs, politicians often see this as a low priority in the face of conflicting demands on the public purse. In both the case of Germany and Japan, a political catalyst was needed to induce the birth of the programs; many countries, including Taiwan and the United States, await their catalysts. Meanwhile, countries developing and adapting programs can learn from LTC programs worldwide.

## Ethical issues

Not applicable.

## Competing interests

Author declares that she has no competing interests.

## Author’s contribution

PN is the single author of the paper.
